# Chemical Composition, Antimicrobial and Antitumor Activities of the Essential Oils and Crude Extracts of *Euphorbia macrorrhiza*

**DOI:** 10.3390/molecules17055030

**Published:** 2012-05-03

**Authors:** Jianbo Lin, Jun Dou, Jiangling Xu, Haji Akber Aisa

**Affiliations:** 1 Key Laboratory of Chemistry of Plant Resources in Arid Regions, Xinjiang Technical Institute of Physics and Chemistry, Chinese Academy of Sciences, Urumqi 830011, China; Email: linjb@ms.xjb.ac.cn (J.L.); doujun68@yahoo.com.cn (J.D.); xujiangling10@mails.gucas.ac.cn (J.X.); 2 Graduate University of the Chinese Academy of Sciences, Beijing 100049, China

**Keywords:** *Euphorbia macrorrhiza*, essential oil composition, antimicrobial activity, antitumor activity

## Abstract

The present study aimed to examine the chemical composition and biological activity of essential oils extracted from *Euphorbia macrorrhiza* collected from Northwest China. The major constituents of the essential oils of aerial parts and roots of *E. macrorrhiza* are acorenone B (16.72% and 25.80%), (+)-cycloisosativene (14.94% and 12.40%), 3a-hydroxy-5b-androstane (10.62% and 5.52%), copaene (7.37% and 6.29%), l-calamenene (4.13% and 4.65%) and β-cedrene (8.40% and 7.98%), respectively. The minor components of them are thymene, γ-terpinene, thymecamphor, α-cedrene, zingiberene, trans-caryophyllene, β-chamigrene, curcumene, pentadecane, (−)-α-muurolene, cuparene, γ-cadinene, (*Z*)-3-heptadecene, 1,3,7,7-tetramethyl-2-oxabicyclo(4.4.0)dec-5-en-4-one, hexahydrofarnesyl acetone, γ-elixene and palmitinic acid. The antimicrobial and antitumor activitiy of the *E. macrorrhiza* essential oil against *Staphyloccocus aureus*, *Escherichia coli*, *Canidia Albicans* and Caco-2 cells were evaluated. Among all the tested microorganisms and Caco-2 cells, the essential oils showed the strongest inhibitory effect on *Staphyloccocus aureus* (MIC = 2.8 μg/mL) and Caco-2 cell (IC_50_= 11.86 μg/mL), whereas no effect on *Escherichia coli* and *Candida albicans*. The data of this study suggested that the *E. macrorrhiza* essential oils have great potential as a natural medicine for microbial infections and cancers.

## 1. Introduction

For thousands of years, humans has gone in different ways to search for cures and relief from various diseases by using numerous plants, plant products and plant-derived products. For some decades now, there has been a considerable interest in screening plant essential oils and extracts for medical use all over the World. As a result, a larger number of these essential oils and crude extracts have shown beneficial therapeutic effects, including anti-oxidant, antimicrobial and antitumor activities [1,2,3,4]. 

The Euphorbiaceae is a large family of the flowering plants, that includes 300 genera and over 5,000 species ranging from annuals to trees [5]. Many species of the genus Euphorbia have been used as medicinal plants in the treatment for skin diseases, gonorrhea, migraine, intestinal parasites and warts [6]. Phytochemical investigation of this genus revealed that many its components are highly bioactive [7,8]. Roots, seeds, latex, stem, stem barks, leaves, and whole plants of the Euphorbia species have been researched. Plants in the Euphorbiaceae family are well known for the chemical diversity of their isoprenoid constituents [9]. Diterpenoids are found in the majority of the genus with many different core frameworks such as jatrophanes, lathyranes, tiglianes, ingenanes, myrsinols, *etc*. [10,11,12,13,14,15,16,17]. Over the past two decades, many biological activities of *Euphorbia* species constituents have been reported. For example, pubescenol, helioscopinolide A, helioscopinolide B, and pubescene D, isolated from *E. pubescens*, showed to be moderate inhibitors for the human tumor cell lines MCF-7 (breast), NCI H460 (lung), and SF-268 (central nervous system). Moreover, helioscopinolide A and helioscopinolide B showed significant activity against *S. aureus* 6538P (2.5 μg/spot) [18]. Three jatrophane diterpenoids isolated from *E. mongolica* had concentration-dependent effects in inhibiting the efflux pump activity of tumor cells in the range 11.2–112 μM [19]. Jolkinolide A from *E. sessiliflora* showed moderate growth inhibition against *M. catarrhalis* at 50 μg/mL concentration [20]. Natarajan *et al*. reported that different extracts of *E. fusiformis* differ significantly in their antibacterial properties and that the methanol extract is the most effective, followed by acetone and chloroform extracts. Especially, rootstock extracts had better antibacterial properties than leaf extracts [21]. The ethanol extracts from aerial parts of *E. hirta* also exhibited broad spectrum of antimicrobial activity against *E. coli*, *P. vulgaris*, *P. aeruginosa*, and *S. aureus* [22].

However, previous work focused in just a few members of this large (over 5,000 species) plant family. To date, not all of the species have been soundly studied seeking potential bioactive components. The plant *Euphorbia macrorrhiza* C. A. Mey is a perennial herb that grows in West Siberia, Kazakhstan and North China. Little has been done about the antitumor, antimicrobial proprieties of *E. macrorrhiza*. This study aimed to investigate the chemical composition of *E. macrorrhiza* essential oils by GC/MS. Besides that the study assessed the antitumor and antimicrobial properties of essential oils and different polarity extracts from aerial parts and roots of *E. macrorrhiza*.

## 2. Results and Discussion

### 2.1. Chemical Composition

**Figure 1:**
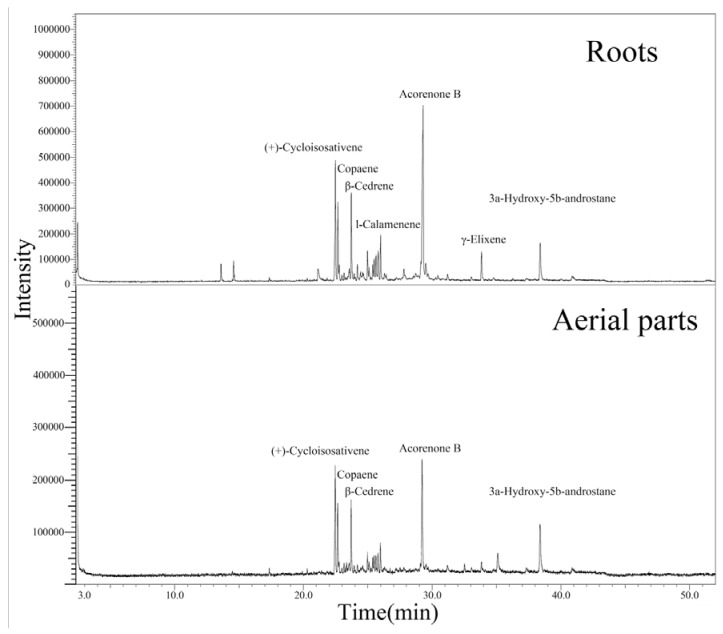
Typical GC/MS chromatograms of the chemical profiles for the *E. macrorrhiza* essential oils.

Our result showed that the *E. macrorrhiza* essential oils contain a complex mixture that consists in its majority of sesquiterpene hydrocarbons. GC-MS analysis of the essential oils led to the identification of 20 and 31 different compounds, representing 88.71 and 97.80% of the total essential oils from aerial parts and roots, respectively. Figure 1 shows the GC-MS profiles of the aerial parts and roots essential oils of *E. macrorrhiza* are very similar. As shown in Table 1, the major compounds detected in the aerial parts and roots essential oils are acorenone B (16.72% and 25.80%), (+)-cycloisosativene (14.94% and 12.40%), 3a-hydroxy-5b-androstane (10.62% and 5.52%), copaene (7.37% and 6.29%), l-calamenene (4.13% and 4.65%) and β-cedrene (8.40% and 7.98%). The minor components are thymene, γ-terpinene, thymecamphor, α-cedrene, zingiberene, *trans*-caryophyllene, β-chamigrene, curcumene, pentadecane, (−)-α-muurolene, cuparene, γ-cadinene, (*Z*)-3-heptadecene, 1,3,7,7-tetramethyl-2-oxabicyclo- (4.4.0)dec-5-en-4-one, hexahydrofarnesyl acetone, γ-elixene and palmitinic acid. Roots essential oil has more compounds by comparison with that from aerial parts.

**Table 1:** Chemical composition of essential oils of *E. macrorrhiza*.

No.	Compounds	RI	Peak area (%)	
			aerial parts	roots
1	Thymene	1025	-	1.49
2	3,7-Dimethyldecane	1056	-	0.14
3	γ-Terpinene	1060	-	1.59
4	Cyclohexyl(dimethoxy)methylsilane	1160	-	0.36
5	Nonane,5-(2-methylpropyl)	1276	0.70	-
6	Thymecamphor	1310	-	2.70
7	(+)-Cycloisosativene	1369	14.94	12.40
8	Copaene	1377	7.37	6.29
9	α-Cedrene	1382	1.29	1.36
10	Octadecyl chloride	1399	-	0.73
11	Valencene	1407	0.93	-
12	Zingiberene	1416	1.46	-
13	β-Cedrene	1423	8.40	7.98
14	Widdrene	1433	0.89	0.47
15	*trans*-Caryophyllene	1444	-	1.54
16	α-Guaiene	1462	-	0.41
17	Bicyclosesquiphellandrene	1464	-	0.26
18	β-Chamigrene	1478	2.41	2.68
19	Curcumene	1483	1.58	1.30
20	Pentadecane	1496	1.58	1.38
21	(−)-α-Muurolene	1501	1.98	1.54
22	Cuparene	1507	1.98	1.83
23	γ-Cadinene	1515	2.89	3.03
24	l-Calamenene	1524	4.13	4.65
25	a-Cadinene	1538	-	0.68
26	Calacorene	1545	-	0.50
27	Humulane-1,6-dien-3-ol	1609	-	0.94
28	Tricyclo[4.4.0.0(2,7)]dec-3-ene-3-	1653	-	0.59
	methanol, 1-methyl-8-(1-methylethyl) -			
29	(*Z*)-3-Heptadecene	1674	-	1.96
30	Acorenone B	1681	16.72	25.8
31	1,3,7,7-Tetramethyl-2-oxa-bicyclo(4.4.0)-dec-5-en-4-one	1691	-	1.66
32	Naphthalene, 1,2,3,4-tetrahydro- 1-methyl-8-(1-methylethyl)-	1776	-	0.87
33	Hexahydrofarnesyl acetone	1841	1.35	-
34	γ-Elixene	1905	1.81	3.67
35	Palmitinic acid	1971	5.68	-
36	3a-Hydroxy-5b-androstane	2146	10.62	5.52
	Total		88.71	97.80

### 2.2. Antimicrobial and Antitumor Activities

In this study, we investigated the antitumor activity against Caco-2 and antimicrobial activity of the essential oils and extracts of different polarity (hexane, chloroform, ethyl acetate, butanol and residual methanol fractions) obtained from *E. macrorrhiza*. The most significant growth inhibitory action was shown by essential oil of roots with an IC_50_ value of 11.86 μg/mL (Table 2). However, no antitumor activities were observed for the different polarity extracts. Evaluation of MIC and MBC/MFC showed that the extracts presented various degrees of inhibition against all the microbes investigated (Table 3). Essential oils and extracts showed various antibacterial activities against *Staphyloccocus aureus*, which could be attributed to the present of sesquiterpene hydrocarbons [23,24]. *Staphyloccocus aureus* strains showed more sensibility to those extracts compared with *Escherichia coli* and *Candida albicans* strains. The value of anti-*Staphyloccocus aureus* activities of the essential oils were higher than that of crude extracts, with MIC values 2.8 μg/mL and 5.6 μg/mL. The essential oils and extracts displayed no antibacterial activity potential with the tested Gram-negative bacteria such as *Escherichia coli*. The Gram-positive bacteria appeared to be more sensitive than Gram-negative to essential oils [25,26].

**Table 2:** Antitumor activity of essential oils and crude extracts of aerial parts and roots of *E. macrorrhiza* for Caco-2 cell.

	Concentration (μg/mL)	Inhibition ratio (%)	IC_50_ (μg/mL)
**Aerial parts**			
EO	250	96.12	78.32
HAF	250	83.57	-
CHF	250	68.22	-
EAF	250	4.61	-
BAF	250	-	-
RMF	250	-	-
**Roots**			
EO	250	96.32	11.86
HAF	250	72.52	-
CHF	250	82.52	-
EAF	250	2.32	-
BAF	250	-	-
RMF	250	-	-

EO: essentials oils; HAF: hexane fraction; CHF: chloroform fraction; EAF: ethyl acetate fraction; BAF: butanol fraction; RMF: residual methanol fractions; IC_50_: half maximal inhibitory concentration.

**Table 3:** Antimicrobial activity (MIC, MBC/MFC) of essential oils and crude extracts from aerial parts and roots of *E. macrorrhiza* for *Staphyloccocus aureus*, *Escherichia coli* and *Canidia albicans* strains. MIC and MBC/MFC were determined by macrodilution method and expressed in μg/mL.

	*Staphyloccocus aureus*		*Escherichia coli*		*Candida* *albicans*	
	MIC	MBC	MIC	MBC	MIC	MFC
**Aerial parts**						
EO	5.6	22.0	>20.0	>20.0	>20.0	>20.0
HAF	1000	2000	>2000	>2000	>2000	>2000
CHF	500	1000	>2000	>2000	>2000	>2000
EAF	1000	1000	>2000	>2000	>2000	>2000
BAF	2000	>2000	>2000	>2000	>2000	>2000
RMF	>2000	>2000	>2000	>2000	>2000	>2000
**Roots**						
EO	2.8	5.6	>20.0	>20.0	>20.0	>20.0
HAF	1000	>2000	>2000	>2000	>2000	>2000
CHF	500	2000	>2000	>2000	>2000	>2000
EAF	500	1000	>2000	>2000	>2000	>2000
BAF	2000	2000	>2000	>2000	>2000	>2000
RMF	>2000	>2000	>2000	>2000	>2000	>2000
Ampicillin	0.25	2.5	>20.0	>20.0	n.t.	n.t.
Amphotericin B	n.t.	n.t.	n.t.	n.t.	0.25	0.25

EO: essentials oils; HAF: hexane fraction; CHF: chloroform fraction; EAF: ethyl acetate fraction; BAF: butanol fraction; RMF: residual methanol fractions; MIC: minimum inhibitory concentration; MFC/MBC: minimum fungicidal/bactericidal concentration; n.t.: not tested.

To sum up, this is the first time to study the chemical composition and bioactivity of *E. macrorrhiza*. Essential oils of *E. macrorrhiza* showed to have useful antimicrobial and antitumor activity.

## 3. Experimental

### 3.1. Plant Materials

The whole plant of *E. macrorrhiza* was collected in 2010 from the Burqin region of Xinjiang (northwest China), and initially identified by Dr. G.M. Shen, Xinjiang Institute of Ecology and Geography, and a voucher specimen has been deposited in Xinjiang Technical Institute of Physics and Chemistry with voucher number 20110009.

### 3.2. Extraction of Essential Oils

The air-dried aerial parts and roots (100 g for each) of the plants were pulverized and subjected to hydrodistillation for 4 h, using a Clevenger-type apparatus. Before analysis and biological activity test, the collected oils were dehydrated with Na_2_SO_4_ and preserved at 4 °C.

### 3.3. Preparation of Crude Extracts

The air-dried aerial parts and roots of *E. macrorrhiza* were pulversized separately. The dried powder (100 g) was extracted three times with methanol (3 × 500 mL) at room temperature and mixed the extracts, then it was evaporated on a vacuum rotary evaporator (Büchi R-210, Flawil, Switzerland). The methanol extract (8.8 g) was suspended in water and extracted successively with hexane, chloroform, ethyl acetate and butanol (3 × 300 mL, 25 °C) to give hexane (2.4 g), chloroform (1.5 g), ethyl acetate (0.6 g), butanol (1.1 g) and residual methanol fractions (2.2 g), respectively.

### 3.4. GC-MS/MS Analysis

GC-MS/MS analysis of the volatile constituents was performed with a Shimadzu GC MS-QP2010 (Kyoto, Japan). RTX-5MS on a fused silica capillary column (30 m × 0.32 mm × 1.0 µm) that was directly coupled to the mass spectrometer. Helium was used as the carrier gas with a flow rate of 1.0 mL/min. The program used was initially 5 min isothermal at 40 °C and then rose to 250 °C at a rate of 5 °C/min, and finally held at that temperature for 5 min. The injection port temperature was 250 °C. One microliter of essential oils sample, dissolved in acetone (1:100, *v*/*v*), was injected. The mass spectrometer was operated in electron impact ionization (EI) mode with 70 eV energy. The mass range was 30–500 Da and the ion source temperature was 200 °C. The individual constituents were identified by their identical retention indices referring to the NIST147 and Wiley7 mass spectra libraries.

### 3.5. Antitumor Activity

Caco-2 cells (human colorectal carcinoma) were obtained from the Cell Bank of the Chinese Academy of Sciences (Shanghai, China). Cells were maintained in Dulbecco’s modified Eagle’s medium (DMEM). When the cells were 80–90% confluent, they were harvested by treatment with a solution containing 0.25% trypsin, thoroughly washed and resuspended in supplemented growth medium. Cells were inoculated into 96-well microtiter plates in 100 µL at plating densities ranging 1.0 × 10^5^ cells/well depending on the doubling time of individual cell lines. After cell inoculation, the microtiter plates were incubated at 37 °C, 5% CO_2_, 95% air and 100% relative humidity. 

The next day, samples were prepared in DMSO, and the concentration ranges tested were 250, 25, 2.5, 0.25 and 0.025 µg/mL. The establishment of five groups of three parallel holes, then 1 μL of corresponding concentration of sample were added to each well.

MTT assay was employed as test for quantification of cell proliferation after 24 h incubation. For this purpose, the medium of each well was removed and 50 μL of MTT solution (1 mg/mL) was added. After 4 h, the medium was removed and the cells were washed with PBS for two times, then replaced with 150 μL of DMSO to dissolve the MTT crystals. The optical density was determined using a Microplate reader SpectraMax M5 (Molecular Devices, Sunnyvale, CA, USA) at a test wavelength of 550 nm. Then the inhibitory percentage of each sample at various concentrations was calculated, and the IC_50_ value was determined. Each data is the mean value of three determinations.

### 3.6. Antimicrobial Activity

Three type strains, *Staphyloccocus aureus* ATCC 6538, *Escherichia coli* ATCC 11229, *Candida albicans* ATCC 10231 were obtained from the National Center for Medical Culture Collections (CMCC), China. All strains were stored at −80 °C in the appropriate medium. Antimicrobial activity of the essential oils and crude extracts was tested using the agar well diffusion method [27]. Luria-Bertani (LB) and Sabaurauds agar (SDA) were sterilised in an autoclave and cooled to 45–50 °C before be poured into 100 mm Petri dishes. The agar plates were stored at 4 °C before used.

*Staphyloccocus aureus* ATCC 6538 and *Escherichia coli* ATCC 11229 were cultured overnight at 37 °C in LB. *Candida albicans* ATCC 10231 was cultured overnight at 37 °C in SDA. Petri dishes with 20 mL of medium were prepared, previously inoculated with 200 μL of the culture suspension. The wells (6 mm) were made and the sample diluted in DMSO to test concentration (100 mg/mL) was added (20 μL/well) and the same volume (20 μL) of DMSO was used as a control. The inoculated plates were incubated for 24/48 h. After incubation, the diameter of the inhibition zone was measured with calipers. 

The minimum inhibitory concentration (MIC) values and the minimum fungicidal/bactericidal concentration (MFC/MBC) values were determined for the microorganisms that were sensitive to the essential oil in the agar well diffusion method. 

The MIC determination was performed by a serial dilution technique, using 96-well microtitre plates. The bacterial inocula applied contained approximately 1.0 × 10^5^ cells in a final volume of 200 μL/well. The extracts tested were dissolved in DMSO (0.01–100 mg/mL) and added to medium with microbial inocula. The microplates were incubated for 24/48 h at 37 °C. The lowest concentrations without visible growth (at the binocular microscope) were defined as concentrations which completely inhibited bacterial growth (MIC). The MFC/MBC were determined by serial subcultivation of 4 μL in microtitre plates containing 196 μL of broth per well and further incubation for 24/48 h at 37 °C. The lowest concentration with no visible growth was defined as the MBC/MFC. DMSO was used as a negative control, while ampicillin and amphotericin B were used as a positive control. Dilutions of the inocula were also cultured on solid LB and SDA to verify the absence of contamination and to check their validity. Each assay was replicated three times.

## 4. Conclusions

Our results demonstrated that *E. macrorrhiza* essential oils displayed significant antimicrobial and antitumor properties. Making this plant a good source for bioactive compounds such as acorenone B, (+)-cycloisosativene, 3a-hydroxy-5b-androstane, copaene, l-calamenene and β-cedrene, which were considered powerful antimicrobial and antitumor agents. We cannot discount the possibility that other minor compounds in the extracts function as bioactive agents or the bioactivity is the result of combination or synergistic effects of some undetermined compounds in the extract in this study. It remains a good natural source for the production of new antimicrobial and antitumor drugs, thereby supporting the broader use of *E. macrorrhiza* in natural medicine for microbial infections and cancer.
